# Endoscopic ultrasonography of a multiseptate gallbladder: differentiation from adenomyomatosis of the gallbladder

**DOI:** 10.1055/a-2599-6956

**Published:** 2025-05-28

**Authors:** Koichi Soga, Fuki Hayakawa, Mayumi Yamaguchi, Takeshi Fujiwara, Ryosaku Shirahashi, Ikuhiro Kobori, Masaya Tamano

**Affiliations:** 1 26263 Department of Gastroenterology, Dokkyo Medical University Saitama Medical Center, Koshigaya, Japan

A multiseptate gallbladder is a rare gallbladder anomaly, with a recorded occurrence of around 150 documented cases globally 1 . Although multiple theories regarding the pathogenesis of multiseptate gallbladder have been proposed, its rarity precludes definitive management guidelines 2 . It is typically diagnosed using imaging, and ultrasonography is considered the most effective and practical diagnostic modality.

We present the case of a 66-year-old Japanese man who was evaluated at our hospital because of pancreatic enlargement and increased IgG4 levels. The patient had been previously diagnosed with adenomyomatosis of the gallbladder neck and had been undergoing long-term observation ( Fig. 1 ). Endoscopic ultrasonography (EUS) of the pancreas demonstrated diffusely reduced echogenicity in the caudal body, which is consistent with chronic progressive autoimmune pancreatitis. EUS of the gallbladder neck identified multiple septations, with a “honeycomb” pattern of linear internal echoes crossing the gallbladder lumen, which are characteristic of multiseptate gallbladder. The gallbladder had a normal size and thin walls; no evidence of wall thickening, pericholecystic fluid, or cholelithiasis was observed. The biliary tract was not significantly dilated. Enhanced EUS images obtained with perflubutane contrast medium were clearer than standard EUS images ( Fig. 2 ; Video 1 ). Based on the EUS findings, we concluded that the diagnosis was multiseptate gallbladder in the gallbladder neck, rather than adenomyomatosis of the gallbladder.

**Fig. 1:**
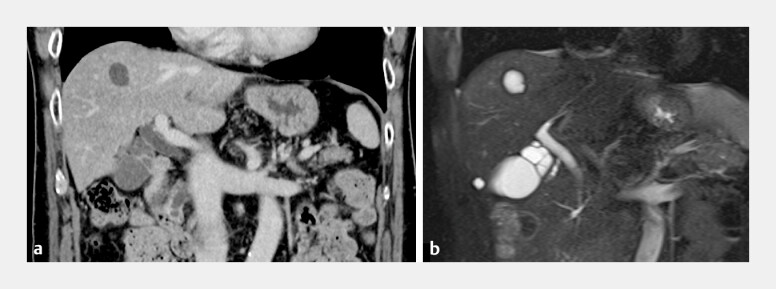
A multiseptate gallbladder that was found incidentally is seen on: **a** contrast-enhanced computed tomography (CT), showing increased contrast enhancement in the affected region, and multiple septations, with a “honeycomb” pattern, in the gallbladder neck; **b** magnetic resonance cholangiopancreatography (MRCP), performed to rule out other abdominal pathologies, showing septations in the gallbladder neck, which are characteristic of a multiseptate gallbladder.

**Fig. 2:**
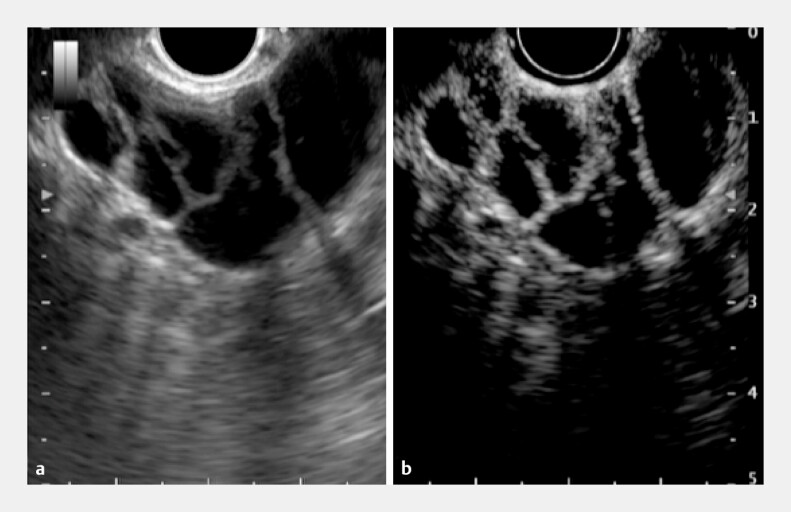
Endoscopic ultrasonography (EUS) images of a multiseptate gallbladder showing: **a** multiple septations in the gallbladder neck that display the typical “honeycomb” pattern of linear internal echoes crossing the gallbladder lumen; the gallbladder is of normal size and has thin walls, with no evidence of wall thickening, pericholecystic fluid, or cholelithiasis, and no significant dilatation of the biliary tract; **b** with perflubutane contrast enhancement, a clearer image of the typical “honeycomb” pattern of linear internal echoes crossing the gallbladder lumen, consistent with the diagnosis of multiseptate gallbladder affecting the gallbladder neck.

Endoscopic ultrasonography of a multiseptate gallbladder showing its differentiation from adenomyomatosis of the gallbladder.Video 1

Because of the limited number of cases, no standardized approach for the evaluation or management of multiseptate gallbladder exists. Conservative management is often appropriate; however, surgical intervention may be performed if other biliary pathologies are present 2 .

Multiseptate gallbladder is a rare anatomical anomaly, with minimal clinical significance as it is generally benign. Symptomatic multiseptate gallbladder can be managed conservatively if associated biliary tree abnormalities are not present. In our patient, the multiseptate gallbladder was an incidental finding that did not require surgical intervention. EUS is useful for diagnosing the condition based on its characteristic findings.

Endoscopy_UCTN_Code_TTT_1AS_2AG

## Notes

Endoscopy E-Videos https://eref.thieme.de/e-videos
*E-Videos* is an open access online section of the journal *Endoscopy* , reporting on interesting cases and new techniques in gastroenterological endoscopy. All papers include a high-quality video and are published with a Creative Commons CC-BY license. Endoscopy E-Videos qualify for HINARI discounts and waivers and eligibility is automatically checked during the submission process. We grant 100% waivers to articles whose corresponding authors are based in Group A countries and 50% waivers to those who are based in Group B countries as classified by Research4Life (see: https://www.research4life.org/access/eligibility/ ). This section has its own submission website at https://mc.manuscriptcentral.com/e-videos .
